# The mediating role of perceived social support between work-family conflict and presenteeism among ICU nurses working shift work in Chinese public hospitals: A cross-sectional investigation

**DOI:** 10.1371/journal.pone.0308673

**Published:** 2024-08-13

**Authors:** Jijun Wu, Yuxin Li, Qin Lin, Yuting Fan, Jiquan Zhang, Zhenfan Liu, Xiaoli Liu, Ping Dai, Xian Rong, Xiaoli Zhong

**Affiliations:** 1 Department of Cardiology, Deyang People’s Hospital, Deyang, Sichuan, China; 2 School of Nursing, North Sichuan Medical College, Nanchong, Sichuan, China; 3 Department of Nursing, Deyang People’s Hospital, Deyang, Sichuan, China; 4 Shulan International Medical College, Zhejiang Shuren University, Hangzhou, Zhejiang, China; 5 School of Nursing, ChengDu College, Chengdu, Sichuan, China; 6 Sichuan Nursing Vocational College, Chengdu, Sichuan, China; Universiti Pertahanan Nasional Malaysia, MALAYSIA

## Abstract

**Objective:**

Relative to explicit absenteeism, nurses’ presenteeism has a more lasting impact and is more harmful and costly. This study aimed to explore the relationship between work-family conflict, perceived social support, and presenteeism and whether perceived social support mediates the relationship between work-family conflict and presenteeism among ICU nurses working on shifts in Chinese public hospitals.

**Materials and methods:**

A cross-sectional research design was conducted from January to April 2023 in Sichuan Province, China. A total of 609 valid questionnaires were collected. The questionnaires contained information on demographic characteristics, the Work-Family Conflict (WFC) scale, the Perceived Social Support Scale (PSSS), and Stanford Presenteeism Scale-6 (SPS-6). Multiple stratified regression was used to explore the mediating role of perceived social support between work-family conflict and presenteeism. The mediating effect of perceived social support in work-family conflict and presenteeism was tested by Model 4 in the PROCESS 4.1 macro program in SPSS.

**Results:**

A total of 609 nurses were included in this study, and the mean presenteeism score for ICU nurses working on shifts was 16.01 ± 4.293 (Mean ± SD), with high presenteeism accounting for 58.46%. After controlling for sociodemographic characteristic variables, work-family conflict was positively associated with presenteeism, explaining 7.7% of the variance. High perceived social support was related to low presenteeism, explaining 11.5% of the variance. Perceived social support mediated the association between work-family conflict and presenteeism among ICU nurses working on shifts.

**Conclusions:**

Chinese shift-work ICU nurses’ high presenteeism scores deserve managers’ attention. Work-family conflict is a significant predictor of nurses’ presenteeism. Perceived social support is essential in improving nurses’ work-family conflict and mediates the relationship between work-family conflict and presenteeism. Improving social support can reduce the impact of work-family conflict on presenteeism among nurses working shifts.

## Introduction

Presenteeism is a typical health-related work productivity deficit in occupational populations, especially in the education and healthcare sectors [1]. Professor Copper introduced the concept of presenteeism in 1996, describing it as a situation in which work productivity is reduced due to illness or long hours at work when one should have taken a break from work at home but still insisted on working [2]. In 2004, Prof. Turpin extended the concept of presenteeism to situations where an individual’s productivity at work is impaired due to health problems [3]. Prof. Turpin et al. extended the idea of presenteeism to include situations where work productivity is impaired due to health problems. A systematic evaluation showed that the prevalence of presenteeism in the working population ranged from 35% to 97% [4]. Due to the global shortage of nursing human resources and the specific nature of nursing, such as high intensity, high stress, shift work, and poor replaceability, the incidence of presenteeism among nurses is four times higher than that of the general corporate workforce. It has been reported that the overall estimated detection rate of presenteeism among nurses worldwide is about 49.2% [5], with 37.4% for nurses in Finland [6], 52.6% for nurses in the United States [7], 55% for nurses in Portugal [8], and as high as 94.25% for nurses in China [9]. Presenteeism can decrease individuals’ subjective well-being, reduce the quality of nursing services, and significantly increase nurses’ willingness to leave their jobs [10,11]. In addition, presenteeism also causes huge economic losses. According to reports, the economic loss of Japanese nurses due to presenteeism is about US$3,055 per year [12], the cost of presenteeism for nurses in the United States is about US$12 billion per year [10], and the cost of presenteeism for nurses in China is about US$4.38 billion per year [9]. Therefore, presenteeism has become one of the most concerning health issues in the global occupational health field.

As an essential place of life support for critically ill patients in medical institutions, the intensive care unit (ICU) has the characteristics of scrambling for time and seconds for rescue, numerous medical equipment, and a high degree of specialization, which leads to heavy workload and more sources of work pressure for nurses [13,14]. China’s ICU nurses work in a cooperative group work mode, the implementation of the three-shift rotation system, commonly known as "three shifts," that is, the whole shift (8:00 ~ 17:00), the night shift (17:00 ~ 00:00) and the next night shift (00:00 ~ 8:00), to ensure the continuity of nursing work 24h [15]. Due to the unique nature of shift work ICU nurses’ working hours and the high workload intensity of ICUs, prolonged shift work not only leads to physiological and psychological health damage and subhealth symptoms but also shift nurses often have work-family conflicts due to the difficulty of balancing work and family, which in turn affects the shift nurses’ work commitment and work efficiency. Coupled with the influence of organizational culture, management decisions, dedication, economic factors, and poor substitutability of shift work positions, the phenomenon of presenteeism of shift work nurses is also particularly prominent [16]. Therefore, focusing on the mechanisms influencing presenteeism of shift work ICU nurses and developing targeted interventions are essential for improving nurses’ physical and mental health and patient safety.

Work and family are the two central areas of life, and work roles and family roles interact, creating a work-family conflict experience when there is an imbalance between the two roles. Work-family conflict occurs when the general demands, time commitment, and pressures of work or family get in the way of fulfilling family or work responsibilities [17]. Studies have shown that 50% of nurses chronically experience disruptions to family caused by work, while 11% experience disruptions to work caused by family life [18,19]. Work-family conflict is a significant factor affecting nurses’ health and work engagement and is significantly associated with nurses’ physical and mental health, organizational commitment, and willingness to leave [20–23]. ICU work is characterized by high intensity, fast pace, frequent emergencies, and relatively insufficient human resources equipped, which, together with the impaired physiological health due to shift work, results in nurses facing more significant psychological pressure and workload pressure. Studies have shown that stress caused by high work demands can lead to physical and psychological problems and work-family conflicts, resulting in presenteeism, such as low work efficiency and reduced work engagement [23–25]. However, presenteeism caused by the conflict between work and family cannot be changed in the short term. Therefore, exploring the influences of mediating variables from the complex relationship and adopting targeted interventions are essential to reduce the impact of work-family conflict on presenteeism. Based on this, we propose research hypothesis 1: work-family conflict positively affects presenteeism, and the effect of work-family conflict on presenteeism can be reduced through mediating variables.

Previous studies on the factors influencing presenteeism among nurses have focused on sociodemographic characteristics, such as length of service and income [26]; health conditions, such as chronic pain, hypertension, gastrointestinal disorders, respiratory disorders, and chronic fatigue [27–30]; and work-related factors, such as occupational stress, and social and work environments, and have often overlooked the potential impact of positive psychological qualities on presenteeism. Therefore, it is essential to explore the relationship between work-family conflict and presenteeism on a positive psychological level and to seek new interventions to prevent the occurrence of presenteeism.

As an important research area in positive psychology, social support emphasizes the impact of interpersonal relationships on mental health and well-being, which can help individuals effectively cope with work stress and maintain a healthy mental state [31]. Perceived social support refers to an individual’s expectation and evaluation of social support, which mainly includes family, friends, and material or spiritual support from society. Perceived level of social support reflects the degree of linkage between individuals and society, and a higher level of social support positively affects individuals’ resistance to stress, depression, and anxiety, enhancing health and well-being [32,33]. Social support and work-family conflict have been widely studied, and their benefits have been confirmed [34]. Currently, there are fewer studies on the mediating effect of social support and presenteeism, and some studies have shown that perceived social support is negatively correlated with presenteeism, i.e., the more social support nurses perceive, the lower the level of presenteeism[35].Specifically, when ICU nurses face greater work pressure, strong social support can not only help them better cope with work pressure and improve their work performance and engagement but also help alleviate their work-family conflicts, thus reducing adverse consequences such as illness and presenteeism. Based on this, we propose research hypothesis 2: Perceived social support is strongly related to presenteeism and hurts presenteeism, and perceived social support mediates the relationship between work-family conflict and presenteeism.

**Fig 1:**
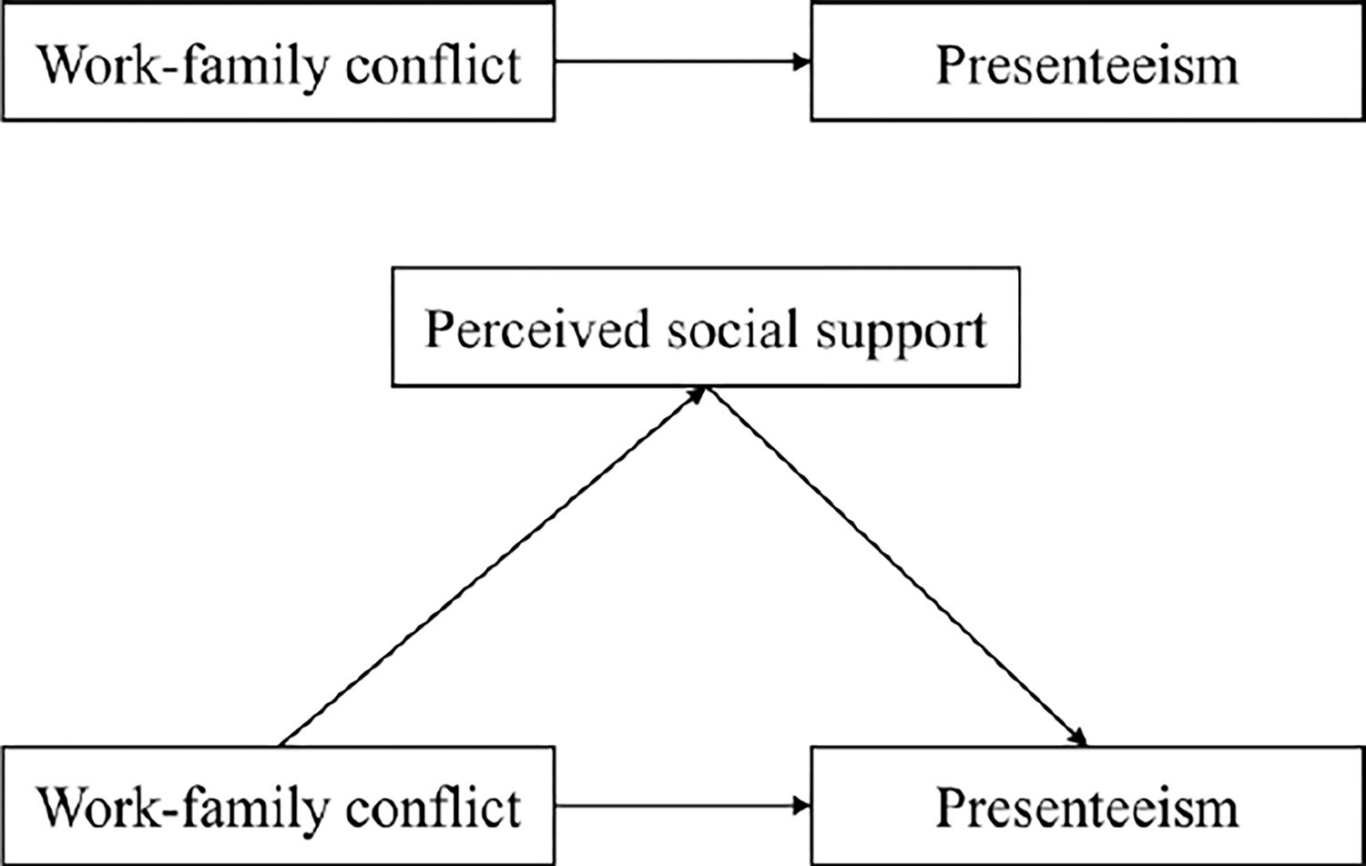
Hypothesized model of the relationship between work-family conflict, perceived social support, and presenteeism.

According to the job demand-resource model [24], job demand refers to the physical, psychological, and social ability requirements of an individual’s job and the factors that require an individual to put in the appropriate effort to complete the job.ICU nurses often have difficulty balancing work and family due to the unique nature of their work. Excessive job demands may exacerbate work-family conflicts among ICU nurses, which affect not only ICU nurses’ mental health but also their work efficiency and quality of care. In addition, work resources refer to factors in work that are related to physiological, psychological, social, or organizational aspects and facilitate the achievement of work goals, reducing work demands and the associated psychophysiological costs. Perceived social support as an adequate work resource: when ICU nurses feel support and help from the surrounding environment, they are more likely to face challenges and difficulties at work with a positive attitude, thus reducing the negative impact of work-family conflict [32]. Based on this, the present study takes the job demand-resource model as the theoretical basis to explore and analyze the effects of work-family conflict on presenteeism among ICU nurses working in shifts from the perspectives of job resources (perceived social support) and job requirements (work-family conflict) and the mediating role of perceived social support between work-family conflict and presenteeism, to provide a theoretical basis for improving nurses’ physical and mental health and decreasing presenteeism. Theoretical basis. The hypothesized model is shown in Fig 1.

## Methods

### Study design and sample

This study was conducted from January to April 2023 in Sichuan Province, China. Sichuan Province is located in southwestern China and has the fourth largest resident population in the country. In this study, two tertiary hospitals were randomly selected from each of the five regions of Sichuan Province (North Sichuan, East Sichuan, South Sichuan, West Sichuan, and Chengdu) using multistage whole cluster random sampling. Each hospital sampled had more than 1,500 beds and more than 1,000 clinical nurses, and a certain percentage of nurses in each region were sampled according to the ICU nurse-patient ratio to make the sample representative. Therefore, in this study, the corresponding proportion of ICU nurses was sampled according to the ratio of 6:2:2 to ensure that the response rate of ICU nurses in each hospital was above 90%. Inclusion criteria: obtaining a professional qualification certificate for nurses, having worked in ICU clinical work for more than one year, having informed consent, and having voluntary participation in this study. Exclusion criteria: those who were currently absent from work on sick leave, maternity leave, or personal leave; internship, regulation training, and conducting nurses.

The number of variables in this study was 21, and according to the Kendall sample size rough estimation method, at least 5–10 times the number of variables were selected, and considering 20% of invalid questionnaires, the sample size ranged from 126 to 252 participants. In this study, the most 685 questionnaires were collected, and 76 questionnaires with apparent regularity of completion (6 or more consecutive entries answered the same way) or omission rate of more than 10% were excluded. 609 shift-work ICU nurses were finally included, with a valid participation rate of 88.91% [36]. The study was conducted in accordance with the Declaration of Helsinki, and the study protocol was approved by the Ethics Committee of Deyang People’s Hospital(2021-04-056-K01). All participants gave informed consent and volunteered to participate in this study.

### Measures

#### Socio-demographic characteristics

Fourteen demographic variables were included in this study: mainly gender, age, marital and childbearing status, et al.

#### Work Family Conflict Scale

A questionnaire developed by Netemeyer et al. was used to measure the perceived level of conflict between nurses’ work and family. The scale consists of two dimensions with ten entries. A 5-point Likert scale was used, with scores ranging from 1 to 5, respectively, from not at all compliant to fully compliant, for a total score of 10 to 50. Higher scores indicated higher levels of work-family conflict faced. In this study, the scale of Cronbach’s α was 0.875 [37].

#### Perceived Social Support Scale

A questionnaire developed by Blumenthal et al. was used. The scale consists of 3 dimensions: family support, friend support, and other support, with 12 entries. A Likert 7-point scale was used, with scores ranging from strongly disagree to strongly agree on a scale of 1 to 7, respectively, for a total score of 12 to 84. Higher scores indicate a higher level of social support available to the individual. The scale of Cronbach’s α was 0.875 [38].

#### Stanford Presenteeism Scale-6

The scale developed by Koopman et al. was used. The scale consisted of two dimensions and six entries. A 5-point Likert scale was used, with scores ranging from 1 to 5 from complete disagreement to complete agreement, respectively, with entries 5 and 6 reversed, for a total score of 6 to 30. Higher scores indicated more severe presenteeism in individuals. The scale of Cronbach’s α was 0.860 [39].

### Procedures

Questionnaire Star was used to create the electronic questionnaire, which was limited to be filled out only once per IP address. After obtaining the consent of the nursing administrators of each hospital, one person in charge of the survey study was selected in each hospital, and all the persons in charge were given unified training to inform them of the purpose, significance, and precautions of this survey study. The person in charge sent the questionnaire link to all nurses’ groups in the hospital, and the first page of the questionnaire was set up with a unified guideline explaining the purpose, content, significance, and precautions for filling out this survey. Following the principle of voluntariness, all those who filled in the questionnaire were regarded as having given their informed consent and could withdraw from the study at any time in the middle. To ensure the completeness of the questionnaire, all options are set as mandatory questions. At the end of the survey, the collected questionnaires were evaluated, and questionnaires with apparent regularity of completion and logical errors were eliminated.

### Statistical analysis

SPSS 26.0 software was used for statistical analysis. The count data were described by frequency and percentage; the measurement data conforming to normal distribution were described by mean ± standard deviation. Independent samples t-test or one-way ANOVA was used to analyze the differences between groups; the Pearson correlation analysis method was used to analyze the correlation between variables, and hierarchical regression analysis was used to analyze the influencing factors of nurses’ hidden absenteeism and the mediating role between variables. Based on the bias-corrected percentile bootstrap method, the mediating role was verified through Model 4 in the PROCESS 4.1 macro program, with work-family conflict as the independent variable, presenteeism as the dependent variable, and perceived social support as the mediating variable.

## Results

### Sociodemographic profiles of shift ICU nurses and relationships with presenteeism, perceived social support, and work-family conflict

Among the 609 ICU nurses working in shifts, 58 were male, and 551 were female; 45.16% were 22~<30 years old, 52.38% were 30 to <40 years old, and 2.46% were ≥40 years old; 196 were unmarried, 402 were married, and 11 were divorced. The rest of the socio-demographic characteristics are shown in Table 1. Socio-demographic information and the relationship with presenteeism, perceived social support, and work-family conflict are shown in Table 2.

**Table 1:** Socio-demographic profile of shift ICU nurses.

Variables	N (%)
Gender	
Male	58 (9.52)
Female	551 (90.48)
Age	
22~<30	275 (45.16)
30~<40	319 (52.38)
≥40	15 (2.46)
Marital or childbearing status	
Unmarried	196 (32.18)
Married with no children	74 (12.15)
Married with children	328 (53.86)
Divorced/widowed	11 (1.81)
Highest education	
Junior college and below	94 (15.44)
Undergraduate	509 (83.58)
Master degree or above	6 (0.98)
Professional title	
Junior ranking	106 (17.41)
Middle level	324 (53.20)
High-level professional title	179 (29.39)
Position	
Clinical Nurse	525 (86.21)
Nursing team leader	71 (11.66)
Head Nurse	13 (2.13)
Years of ICU work	
1~<5	259 (42.53)
5~<10	212 (34.81)
≥10	138 (22.66)
Income per month	
1~<6000	155 (25.45)
6000~<8000	275 (45.16)
8000~<10000	137 (22.50)
≥10000	42 (6.89)
Type of contract	
Professional preparation	92 (15.11)
Labor contract	517 (84.89)
Self-assessed health status	
Good	339 (55.67)
General	239 (38.24)
Worse	31 (5.09)
Suffering from chronic disease	
No	515 (84.56)
Yes	94 (15.44)
Perceived work stress	
Low	13 (2.13)
Moderate	301 (49.44)
High	295 (48.44)
ICU human resource allocation	
<1:2.5~3	357 (58.62)
= 1:2.5~3	148 (24.30)
>1:2.5~3	104 (17.08)
Experienced workplace violence in the past year	
No	399 (77.00)
Yes	210 (33.00)

**Table 2:** Sociodemographic profiles of shift ICU nurses and relationships with presenteeism, perceived social support, and work-family conflict.

Variables	Mean ±SD		
	Presenteeism	Perceived social support	Work-family conflict
Gender			
Male	15.64±4.86	64.48±12.26	26.17±9.76
Female	16.05±4.23	62.31±12.11	27.57±7.55
t	-0.702	1.298	-1.302
*P*	0.48	0.20	0.19
Age			
22~<30	15.92±4.08	63.48±11.71	26.39±8.17
30~<40	16.09±4.56	61.85±12.45	28.27±7.42
≥40	16.07±1.62	59.00±11.81	29.00±6.33
F	0.122	1.892	4.697
*P*	0.89	0.14	0.01
Marital or childbearing status			
Unmarried	16.40±4.14	62.24±12.02	26.66±8.09
Married with no children	15.99±3.87	63.45±11.10	26.55±7.36
Married with children	15.76±4.48	63.27±15.69	28.14±7.66
Divorced/widowed	16.82±3.97	62.52±12.13	26.36±8.26
F	1.042	0.196	1.938
*P*	0.37	0.90	0.12
Highest education			
Junior college and below	15.90±4.12	64.29±11.35	25.67±9.21
Undergraduate	16.04±4.33	62.21±12.22	27.73±7.45
Master degree or above	15.50±4.46	60.67±15.48	30.50±8.98
F	0.084	1.233	3.261
*P*	0.92	0.29	0.04
Professional title			
Junior ranking	16.00±4.31	62.56±12.42	25.85±9.02
Middle level	16.21±4.16	62.29±12.23	27.65±7.38
High-level professional title	15.67±4.52	62.91±11.83	27.99±7.67
F	0.911	0.148	2.792
*P*	0.40	0.86	0.06
Position			
Clinical Nurse	16.10±4.27	62.32±12.35	27.26±7.78
Nursing team leader	15.58±4.61	64.25±10.83	28.59±8.02
Head Nurse	14.85±3.36	60.85±9.56	28.46±6.99
F	0.960	0.917	1.032
*P*	0.38	0.40	0.36
Years of ICU work			
1~<5	15.55±4.17	63.60±12.27	26.57±8.22
5~<10	16.54±4.21	61.33±12.06	27.73±7.30
≥10	16.09±4.58	62.31±11.87	28.62±7.57
F	3.143	2.072	3.354
*P*	0.04	0.13	0.04
Income per month			
1~<6000	16.34±4.21	60.39±13.05	26.61±8.14
6000~<8000	16.12±4.43	62.43±11.93	27.60±7.56
8000~<10000	15.51±4.08	64.53±11.57	27.76±8.10
≥10000	15.76±4.39	64.36±10.57	28.38±6.92
F	1.025	3.197	0.901
*P*	0.38	0.02	0.44
Type of contract			
Professional preparation	15.46±3.76	64.03±9.89	28.79±7.11
Labor contract	16.11±4.38	62.25±12.47	27.20±7.89
t	-1.355	1.301	1.814
*P*	0.18	0.19	0.07
Self-assessed health status			
Good	14.87±4.16	65.14±11.48	25.63±7.90
General	17.08±3.90	59.95±11.60	29.37±6.93
Worse	20.35±3.83	53.61±14.69	32.35±7.48
F	39.651	23.228	24.459
*P*	<0.001	<0.001	<0.001
Suffering from chronic disease			
No	15.80±4.30	63.14±11.91	27.04±7.83
Yes	17.17±4.07	59.13±12.82	29.62±7.24
t	-2.854	2.965	-2.967
*P*	0.04	0.03	0.03
Perceived work stress			
Low	12.38±3.89	72.23±13.06	18.62±9.47
Moderate	14.89±3.81	64.39±11.17	25.24±7.61
High	17.32±4.37	60.18±12.52	30.07±6.89
F	31.361	13.820	42.091
*P*	<0.001	<0.001	<0.001
ICU human resource allocation			
<1:2.5~3	15.75±4.46	62.47±12.17	27.10±8.03
= 1:2.5~3	16.14±3.87	62.32±12.37	16.91±7.43
>1:2.5~3	16.74±4.25	62.95±11.75	29.37±7.24
F	2.235	0.089	3.901
*P*	0.11	0.92	0.02
Experienced workplace violence in the past year			
No	15.30±4.28	63.85±12.05	25.89±7.68
Yes	17.37±3.99	59.99±11.91	30.38±7.14
t	-5.808	3.770	-7.024
*P*	<0.001	<0.001	<0.001

### Descriptive statistics and correlations between variables of work-family conflict, perceived social support, and presenteeism in shift work ICU nurses

Shift work ICU nurse’s work-family conflict score was (27.44±7.79), the perceived social support score was (62.52±12.13), and the presenteeism score was (16.01±4.29).Work-family conflict was positively correlated with presenteeism (r = 0.439, *P*<0.05), work-family conflict was negatively correlated with perceived social support (r = -0.348, *P*<0.05), and comprehending social support was negatively correlated with presenteeism (r = -0.383, *P*<0.05) See Table 3.

**Table 3:** Correlation between work-family conflict, perceived social support, and presenteeism.

Variables	1	2	3
(1) Work-family conflict	1		
(2) Perceived social support	-0.348*	1	
(3) Presenteeism	0.439*	-0.383*	1

* *P* <0.05.

### Multi-stratified regression results

In the first step of the multivariate hierarchical regression, the meaningful variables in the univariate analysis of presenteeism were added to the model as control variables. After excluding the effect of the above control variables in the second step, work-family conflict had a significant impact on presenteeism, explaining 7.7% of the variance. In the third step, adding the mediating variable of perceived social support to the model explains another 11.5% of the variance in presenteeism. The regression coefficient for work-family conflict decreased from 0.312 in the second step to 0.255 in the third step, which is still significant. Statistical analyses initially showed that perceptual social support partially mediated the relationship between work-family conflict and presenteeism among ICU nurses working on shifts. See Table 4.

**Table 4:** Multivariate stratified regression analysis of presenteeism.

Variables	Model 1			Model 2			Model 3		
	*β*	*t*	*P*	*β*	*t*	*P*	*β*	*t*	*P*
Control variables									
Years of ICU work	0.056	1.524	0.128	0.028	0.790	0.430	0.023	0.669	0.504
Self-assessed health status	0.260	6.522	<0.001	0.208	5.386	<0.001	0.173	4.536	<0.001
Suffering from chronic disease	0.007	-0.182	0.856	0.010	0.278	0.781	0.016	0.444	0.657
Perceived work stress	0.216	5.635	<0.001	0.132	3.477	0.001	0.118	3.190	0.001
Experienced workplace violence in the past year	-0.141	-3.693	<0.001	-0.082	-2.203	0.028	-0.076	-2.089	0.037
Work-family conflict				0.312	7.993	<0.001	0.255	6.487	<0.001
Perceived social support							-0.214	-5.759	<0.001
*F* (*P*-value)		28.094 (<0.001)			36.501 (<0.001)			37.696 (<0.001)	
*R*^2^		0.189			0.267			0.305	
*R*^2^ change		0.182			0.259			0.297	

* *P* <0.05

** *P* <0.01.

### Perceived social support mediates the relationship between work-family conflict and presenteeism in shift work ICU nurses

**Fig 2:**
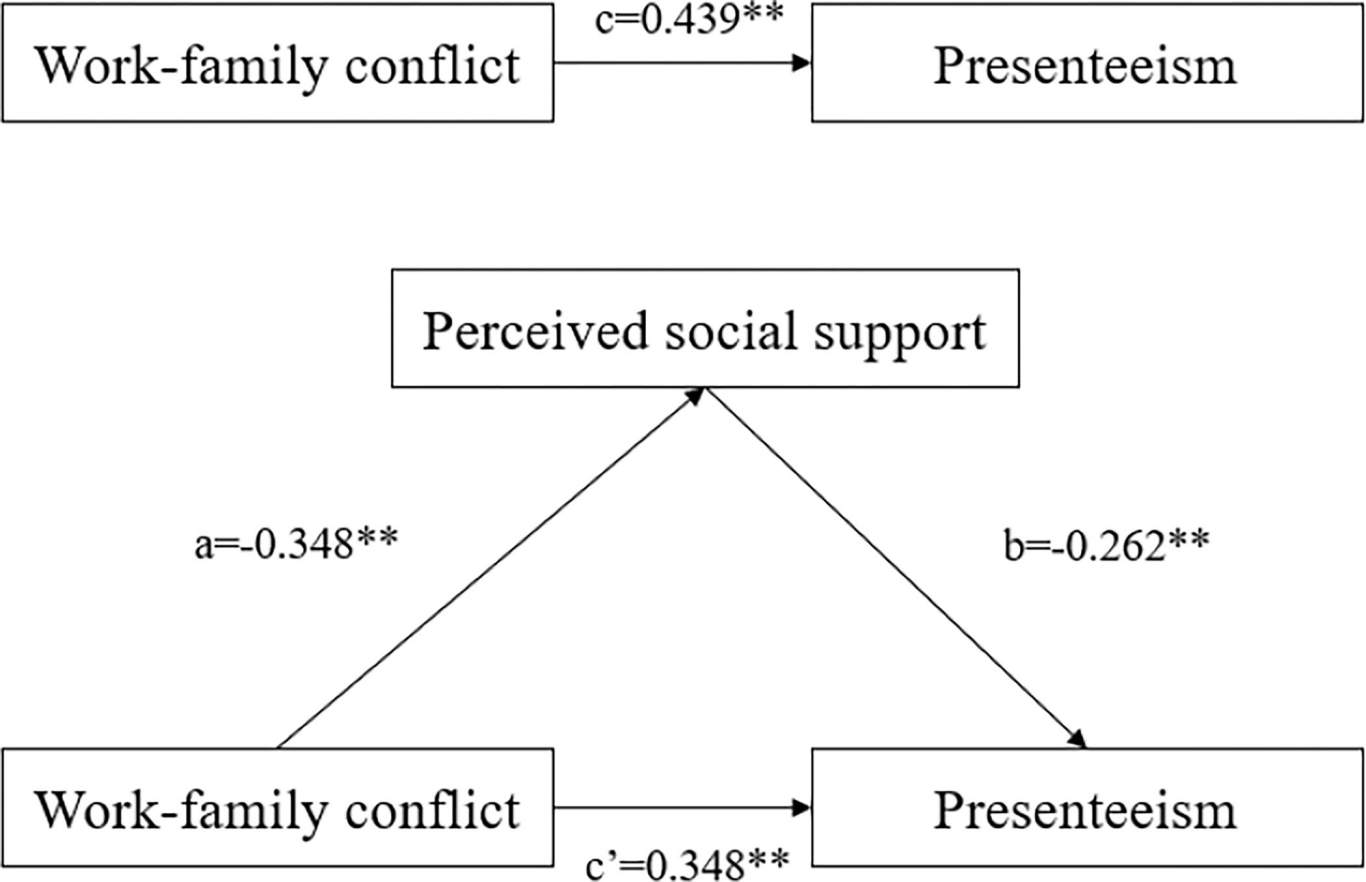
Model of the mediating role of work-family conflict and presenteeism.

First, we analyzed the relationship between work-family conflict and presenteeism (path c). Work-family conflict was positively related to presenteeism, and work-family conflict had a significant indirect influence on presenteeism through perceived social support. The 95% CI confidence interval for the indirect effect did not contain 0, suggesting that perceived social support mediates the relationship between work-family conflict and presenteeism. See Table 5 and Fig 2.

**Table 5:** Results of the analysis of mediating roles.

Path	Coefficient/ Effect	*P*	95% *CI*
c	0.439	<0.01	(0.367, 0.510)
a	-0.348	<0.01	(-0.423, -0.273)
b	-0.262	<0.01	(-0.336, -0.189)
a*b	0.096	-	-
c’	0.348	<0.01	(0.274, 0.421)

## Discussion

This study explored the relationship between work-family conflict, perceived social support, and presenteeism among ICU nurses working shifts. It also analyzed the mediating role of perceived social support between work-family conflict and presenteeism. It was found that work-family conflict negatively affected presenteeism, and perceived social support was a protective factor for presenteeism, in which perceived social support partially mediated between it. In this study, the mean presenteeism score of ICU nurses working shifts was 16.01 ± 4.29 (Mean ± SD), of which high presenteeism accounted for 58.46%, which was higher than the presenteeism rate of nurses with the global rate of nurses (49.2%) [5], the rate of Portuguese nurses with the presenteeism rate of Portuguese nurses (55%) [8], the rate of US nurses with the presenteeism rate of American nurses (52.65%) [7], and higher than the rate of China’s ICU nurses with the presenteeism rate of Chinese nurses (55.4%) [40], suggesting a higher level of presenteeism among ICU nurses working in shifts in China. Studies have shown that shift work significantly impacts nurses’ physiological and psychological health, and shift work nurses tend to report higher levels of subhealth symptoms than day shift nurses, suggesting that attention should be focused on shift nurses’ presenteeism.

First, a positive correlation exists between work-family conflict and presenteeism, consistent with related studies [41]. Studies have shown that work and family are crucial areas of life that require individuals to invest time, feelings, and behaviors. When the needs of the two regions cannot be met simultaneously, and the balance is disturbed, the conflict between work and family arises [42]. Nurses, most of whom are predominantly female, have diverse roles as health caregivers, health educators, and health counselors in their professions. In the family, they also have to take on a variety of family role functions such as daughter, wife, mother, etc. A conflict between the needs of multiple roles will cause a certain amount of psychological pressure and psychological burden to the individual, which can develop into hidden absenteeism. Presenteeism leads to the inability of nurses to maintain their normal working conditions, which not only reduces work efficiency but also leads to an increase in work errors and adversely affects patient safety [43]. High levels of work-family conflict result in nurses being unable to balance their role functioning between work and family, resulting in interactive work-family role conflict, leading to decreased work engagement and, thus, presenteeism [44]. Additionally, shift nurses with high levels of presenteeism are at higher risk for work-family conflict because the accompanying decline in health productivity leads to increased stress in the nurses’ work and requires more time and energy, thereby increasing their impact on family life. In addition, prolonged periods of presenteeism may lead to increased mental health problems and negative emotions such as frustration, helplessness, and anxiety among nurses, exposing them to more psychological distress and an inability to realize the demands of their work-family roles in a high-quality manner, with a corresponding increase in work-family conflict [45]. This study aimed to explore the impact of mitigating work-family conflict on presenteeism, and it was found that navigating social support may fill this gap.

Perceived social support, as a resource of exogenous origin, is a stable psychological trait widely defined as beliefs about the accessibility and satisfaction of social support. Compared to objective social support, perceived social support is more critical for an individual’s mental health, reducing negative psychological traits and thus increasing psychological resilience and adjustment to stressful situations [33,46]. In this study, perceived social support was negatively related to presenteeism, consistent with related studies [35]. Indeed, high levels of perceptual social support are a resilience protective factor for individuals to cope effectively with their family and work roles. Individuals with higher levels of perceptual social support have access to more exogenous supportive resources in stressful environments, thus realizing a buffer against role conflict and, to a certain extent, avoiding the damage of undesirable factors [47]. In contrast, people with lower levels of perceived social support are more likely to be self-denying and adopt withdrawal and avoidance coping styles during stress and role conflict, thus experiencing presenteeism that prevents them from effectively engaging in their work, resulting in decreased work efficiency and reduced work engagement. Previous research has also confirmed that perceived social support positively affects individual work engagement.

The present study confirmed that perceived social support mediates the relationship between work-family conflict and presenteeism among ICU nurses working on shifts. While previous studies have focused more on the effect of perceived social support on work-family conflict [34,48–50], our study found that higher work-family conflict may decrease perceived social support, leading to increased presenteeism. Perceived social support is a cognitive schema that can explain how individuals effectively draw on external support to buffer stressful events and reduce negative psychological traits, reflecting their perception of social relationships. In this case, individuals can achieve work-family role balance through access to social support, thereby reducing work-family conflict and presenteeism. Although this finding is scarce in the literature on shift-work nurses, the theory suggests that perceived social support can partially mediate work-family conflict and reduce work productivity and engagement. To analyze why: work-family conflict may reduce individuals’ social support accessibility and satisfaction because they perceive that they do not have sufficient exogenous resources to balance their work and family roles and are not able to be fully engaged in their work [51,52]. Shift work nurses are required to rotate between day and night shifts to fulfill their work assignments due to the work hour system, which results in irregular work hours and the need for nurses to work during the day, at night, and even on weekends, which can lead to a lack of family role functioning and negatively impact family life [53,54]. In addition, shift work itself faces high work intensity, psychological stress, and disruption of circadian rhythms, coupled with heavy workloads and high risks in ICUs as centrally located admission centers for critically ill patients, which is a high incidence of impaired physiological and psychological health. These studies show that work-family conflict and physical and mental health problems are prominent among ICU nurses working shifts, and we can reduce nurses’ work-family role conflict and presenteeism due to physical and mental health by enhancing social support.

Based on the results of this study, we put forward the following suggestions to improve the hidden absenteeism of ICU nurses working in shifts. First, the state vigorously develops nursing education. It increases the cultivation of nursing talents, especially nursing talents with high educational levels, to alleviate the shortage of nursing human resources, reduce the workload of nurses, and improve their physical and mental health; second, improve the working environment of nursing staff, minimize work pressure, pay attention to the health status of nurses working in shifts, reduce the high-frequency of shifts, and give them sufficient leave after night shifts. They are third, avoiding long shifts and helping nurses find the best balance between shift work and health to reduce the impact of shift-related illnesses. They also enable nurses to seek professional help when facing health problems. Fourth, managers should emphasize the balance between nurses’ family and work roles and reduce extra work outside of work hours to reduce the impairment of nurses’ physical and mental health caused by work-family conflicts. Fourth, given that perceived social support plays a vital role in work-family conflict and hidden absenteeism among ICU nurses working in shifts, we should strengthen the positive publicity of nurses’ professional image, create a good social atmosphere, increase the respect and recognition of the nursing profession in the society, and encourage nurses to set up mutual aid organizations among themselves to support and help each other to cope with the challenges and difficulties of the workplace. In addition, unit managers should formulate scientific and reasonable scheduling principles according to the environmental characteristics of each unit, patients’ conditions, nursing workload, etc., to effectively utilize human resources, improve nurses’ job satisfaction, and reduce presenteeism.

### Limitation

First, this study used a cross-sectional study, which cannot explore causal relationships between variables. Second, this study’s presenteeism mainly reflected the level of relevant events that occurred in the past month of the participants, which may have a recall bias. In addition, only ten randomly selected nurses working in ICU shifts in Sichuan Province, China, were included in this study. The next step is to conduct a multicenter study to expand the survey population and explore the characteristics of presenteeism and the factors that influence nurses working in ICU shifts in hospitals at different levels and in other regions with various economic developments.

## Conclusions

Research on presenteeism of employees has just started in China, mainly focusing on the incidence of presenteeism and the analysis of influencing factors. As a common problem among medical staff, work-family conflict is closely related to work efficiency and engagement. Still, research is lacking in China, especially among the particular group of shift-work nurses. This study was the first to investigate the effects of work-family conflict and perceived social support on presenteeism among ICU nurses working in shifts and to analyze the mediating role of comprehension social support. It is suggested that measures related to reducing work-family conflict and enhancing perceived social support among shift-work nurses may minimize the occurrence of presenteeism among nurses.

## Supporting information

S1 Raw data (XLSX)

## References

[1] Aronsson G, Gustafsson K, Dallner M. Sick but yet at work. An empirical study of sickness presenteeism. J Epidemiol Community Health. 2000;54(7):502–509. doi: 10.1136/jech.54.7.502 10846192 PMC1731716

[2] Chapman LS. Presenteeism and its role in worksite health promotion. Am J Health Promot. 2005;19(4):suppl 1–8. 15768928

[3] Turpin RS, Ozminkowski RJ, Sharda CE, Collins JJ, Berger ML, Billotti GM, et al. Reliability and validity of the Stanford Presenteeism Scale. J Occup Environ Med. 2004;46(11):1123–1133. doi: 10.1097/01.jom.0000144999.35675.a0 15534499

[4] Webster RK, Liu R, Karimullina K, Hall I, Amlôt R, Rubin GJ. A systematic review of infectious illness Presenteeism: prevalence, reasons and risk factors. BMC Public Health. 2019;19(1):799. doi: 10.1186/s12889-019-7138-x 31226966 PMC6588911

[5] Min A, Kang M, Park H. Global prevalence of presenteeism in the nursing workforce: A meta-analysis of 28 studies from 14 countries. J Nurs Manag. 2022;30(7):2811–2824. doi: 10.1111/jonm.13688 35593655

[6] Rantanen I, Tuominen R. Relative magnitude of presenteeism and absenteeism and work-related factors affecting them among health care professionals. Int Arch Occup Environ Health. 2011;84(2):225–230. doi: 10.1007/s00420-010-0604-5 21140162

[7] Warren CL, White-Means SI, Wicks MN, Chang CF, Gourley D, Rice M. Cost burden of the presenteeism health outcome: diverse workforce of nurses and pharmacists. J Occup Environ Med. 2011;53(1):90–99. doi: 10.1097/JOM.0b013e3182028d38 21187792

[8] Mosteiro-Díaz MP, Baldonedo-Mosteiro M, Borges E, Baptista P, Queirós C, Sánchez-Zaballos M, et al. Presenteeism in nurses: comparative study of Spanish, Portuguese and Brazilian nurses. Int Nurs Rev. 2020;67(4):466–475. doi: 10.1111/inr.12615 32844446

[9] Shan G, Wang S, Wang W, Guo S, Li Y. Presenteeism in Nurses: Prevalence, Consequences, and Causes From the Perspectives of Nurses and Chief Nurses. Front Psychiatry. 2020;11:584040. doi: 10.3389/fpsyt.2020.584040 33488418 PMC7819974

[10] Letvak SA, Ruhm CJ, Gupta SN. Nurses’ presenteeism and its effects on self-reported quality of care and costs. Am J Nurs. 2012;112(2):30–38; quiz 48, 39. doi: 10.1097/01.NAJ.0000411176.15696.f9 22261652

[11] Ren Z, Sun Y, Li X, He M, Shi H, Zhao H, et al. How Do Presenteeism and Family Functioning Affect the Association Between Chinese Nurses’ Job Stress and Intention to Stay? J Am Psychiatr Nurses Assoc. 2022:10783903221140329. doi: 10.1177/10783903221140329 36457173

[12] Nagata T, Mori K, Ohtani M, Nagata M, Kajiki S, Fujino Y, et al. Total Health-Related Costs Due to Absenteeism, Presenteeism, and Medical and Pharmaceutical Expenses in Japanese Employers. J Occup Environ Med. 2018;60(5):e273–e280. doi: 10.1097/JOM.0000000000001291 29394196 PMC5959215

[13] Welp A, Rothen HU, Massarotto P, Manser T. Teamwork and clinician burnout in Swiss intensive care: the predictive role of workload, and demographic and unit characteristics. Swiss Med Wkly. 2019;149:w20033. doi: 10.4414/smw.2019.20033 30905060

[14] Aragão NSC, Barbosa GB, Santos CLC, Nascimento D, Bôas L, Martins Júnior DF, et al. Burnout Syndrome and Associated Factors in Intensive Care Unit Nurses. Rev Bras Enferm. 2021;74(suppl 3):e20190535. doi: 10.1590/0034-7167-2019-0535 33503204

[15] Zhang S., Ma H.M., &Shen X.Y. Effectiveness of APN scheduling model in clinical nursing work:a Meta-analysis.Chin Nurs Manag 14,834–838.

[16] Min A, Kang M, Hong HC. Sickness Presenteeism in Shift and Non-Shift Nurses: Using the Fifth Korean Working Conditions Survey. Int J Environ Res Public Health. 2021;18(6). doi: 10.3390/ijerph18063236 33800982 PMC8004057

[17] Borgmann LS, Rattay P, Lampert T. Health-Related Consequences of Work-Family Conflict From a European Perspective: Results of a Scoping Review. Front Public Health. 2019;7:189. doi: 10.3389/fpubh.2019.00189 31338358 PMC6629821

[18] Zhang Y, Duffy JF, De Castillero ER. Do sleep disturbances mediate the association between work-family conflict and depressive symptoms among nurses? A cross-sectional study. J Psychiatr Ment Health Nurs. 2017;24(8):620–628. doi: 10.1111/jpm.12409 28635074 PMC5585039

[19] Grzywacz JG, Frone MR, Brewer CS, Kovner CT. Quantifying work-family conflict among registered nurses. Res Nurs Health. 2006;29(5):414–426. doi: 10.1002/nur.20133 16977647

[20] Hatam N, Jalali MT, Askarian M, Kharazmi E. Relationship between Family-Work and Work-Family Conflict with Organizational Commitment and Desertion Intention among Nurses and Paramedical Staff at Hospitals. Int J Community Based Nurs Midwifery. 2016;4(2):107–118. 27218108 PMC4876779

[21] Matsuo M, Suzuki E, Takayama Y, Shibata S, Sato K. Influence of Striving for Work-Life Balance and Sense of Coherence on Intention to Leave Among Nurses: A 6-Month Prospective Survey. Inquiry. 2021;58:469580211005192. doi: 10.1177/00469580211005192 33769128 PMC8743965

[22] Berkman LF, Liu SY, Hammer L, Moen P, Klein LC, Kelly E, et al. Work-family conflict, cardiometabolic risk, and sleep duration in nursing employees. J Occup Health Psychol. 2015;20(4):420–433. doi: 10.1037/a0039143 25961758 PMC4586296

[23] Nilsen W, Skipstein A, Østby KA, Mykletun A. Examination of the double burden hypothesis-a systematic review of work-family conflict and sickness absence. Eur J Public Health. 2017;27(3):465–471. doi: 10.1093/eurpub/ckx054 28486653 PMC5445721

[24] Demerouti E, Bakker AB, Nachreiner F, Schaufeli WB. The job demands-resources model of burnout. J. Appl. Psychol. 2001, 86: 499–512. 11419809

[25] Holland P, Tham TL, Sheehan C, Cooper B. The impact of perceived workload on nurse satisfaction with work-life balance and intention to leave the occupation. Appl Nurs Res. 2019;49:70–76. doi: 10.1016/j.apnr.2019.06.001 31375315

[26] Li Y, Guo B, Wang Y, Lv X, Li R, Guan X, et al. Serial-Multiple Mediation of Job Burnout and Fatigue in the Relationship Between Sickness Presenteeism and Productivity Loss in Nurses: A Multicenter Cross-Sectional Study. Front Public Health. 2021;9:812737. doi: 10.3389/fpubh.2021.812737 35096756 PMC8795673

[27] Brborović H, Daka Q, Dakaj K, Brborović O. Antecedents and associations of sickness presenteeism and sickness absenteeism in nurses: A systematic review. Int J Nurs Pract. 2017;23(6). doi: 10.1111/ijn.12598 29094426

[28] Fiorini LA, Houdmont J, Griffiths A. Nurses’ illness perceptions during presenteeism and absenteeism. Occup Med (Lond). 2020;70(2):101–106. doi: 10.1093/occmed/kqaa012 31961931

[29] Yoshimoto T, Oka H, Ochiai H, Ishikawa S, Kokaze A, Muranaga S, et al. Presenteeism and Associated Factors Among Nursing Personnel with Low Back Pain: A Cross-Sectional Study. J Pain Res. 2020;13:2979–2986. doi: 10.2147/JPR.S269529 33239906 PMC7682615

[30] Kuster SP, Böni J, Kouyos RD, Huber M, Schmutz S, Shah C, et al. Absenteeism and presenteeism in healthcare workers due to respiratory illness. Infect Control Hosp Epidemiol. 2021;42(3):268–273. doi: 10.1017/ice.2020.444 33239124

[31] Lakey B, Orehek E. Relational regulation theory: a new approach to explain the link between perceived social support and mental health. Psychol Rev. 2011;118(3):482–495. doi: 10.1037/a0023477 21534704

[32] Liu Y, Aungsuroch Y. Work stress, perceived social support, self-efficacy and burnout among Chinese registered nurses. J Nurs Manag. 2019;27(7):1445–1453. doi: 10.1111/jonm.12828 31306524

[33] Öksüz E, Demiralp M, Mersin S, Tüzer H, Aksu M, Sarıkoc G. Resilience in nurses in terms of perceived social support, job satisfaction and certain variables. J Nurs Manag. 2019;27(2):423–432. doi: 10.1111/jonm.12703 30209847

[34] French KA, Dumani S, Allen TD, Shockley KM. A meta-analysis of work-family conflict and social support. Psychol Bull. 2018;144(3):284–314. doi: 10.1037/bul0000120 29239632 PMC5858956

[35] Yang T, Ma T, Liu P, Liu Y, Chen Q, Guo Y, et al. Perceived social support and presenteeism among healthcare workers in China: the mediating role of organizational commitment. Environ Health Prev Med. 2019;24(1):55. doi: 10.1186/s12199-019-0814-8 31481032 PMC6724257

[36] Ni P, Chen JL, Liu N. The sample size estimation in quantitative nursing research. Chin. J. Nurs. 2010, 45, 378–380.

[37] Netemeyer RG, Boles JS, McMurrian R. Development and Validation of Work−Family Conflict and Family−Work Conflict Scales. Journal of Applied Psychology. 1996, 81 (4): 400–410.

[38] Blumenthal JA, Burg MM, Barefoot J, Williams RB, Haney T, Zimet G. Social support, type A behavior, and coronary artery disease. Psychosom Med. 1987;49(4):331–340. doi: 10.1097/00006842-198707000-00002 3615762

[39] Koopman C, Pelletier KR, Murray JF, Sharda CE, Berger ML, Turpin RS, et al. Stanford presenteeism scale: health status and employee productivity. J Occup Environ Med. 2002;44(1):14–20. doi: 10.1097/00043764-200201000-00004 11802460

[40] Zhang Y., Lei S., Chen L. & Yang F. Influence of job demands on implicit absenteeism in Chinese nurses: mediating effects of work-family conflict and job embeddedness. Front Psychol 14, 1265710. doi: 10.3389/fpsyg.2023.1265710 37936572 PMC10627013

[41] Zhang Y, Lei S, Chen L, Yang F. Influence of job demands on implicit absenteeism in Chinese nurses: mediating effects of work-family conflict and job embeddedness. Front Psychol. 2023;14:1265710. doi: 10.3389/fpsyg.2023.1265710 37936572 PMC10627013

[42] Labrague LJ, Ballad CA, Fronda DC. Predictors and outcomes of work-family conflict among nurses. Int Nurs Rev. 2021;68(3):349–357. doi: 10.1111/inr.12642 33165960

[43] Wang X, Qin H, Zhu Y, Wang Z, Ye B, Zhu X, et al. Association of off-the-job training with work performance and work-family conflict among physicians: a cross-sectional study in China. BMJ Open. 2022;12(1):e053280. doi: 10.1136/bmjopen-2021-053280 35017246 PMC8753420

[44] Gillet N, Austin S, Fernet C, Sandrin E, Lorho F, Brault S, et al. Workaholism, presenteeism, work-family conflicts and personal and work outcomes: Testing a moderated mediation model. J Clin Nurs. 2021;30(19–20):2842–2853. doi: 10.1111/jocn.15791 33870550

[45] Xi X, Lu Q, Wo T, Pei P, Lin G, Hu H, et al. Doctor’s presenteeism and its relationship with anxiety and depression: a cross-sectional survey study in China. BMJ Open. 2019;9(7):e028844. doi: 10.1136/bmjopen-2018-028844 31366652 PMC6677964

[46] Yan J, Wu C, He C, Lin Y, He S, Du Y, et al. The social support, psychological resilience and quality of life of nurses in infectious disease departments in China: A mediated model. J Nurs Manag. 2022;30(8):4503–4513. doi: 10.1111/jonm.13889 36325798 PMC9878052

[47] Chen JW, Lu L, Cooper CL. The Compensatory Protective Effects of Social Support at Work in Presenteeism During the Coronavirus Disease Pandemic. Front Psychol. 2021;12:643437. doi: 10.3389/fpsyg.2021.643437 33833723 PMC8021870

[48] Li C, Song R, Fan X, Zhou Z, Xu L. Moderating effect of work-related social support on the relationship between role conflicts and job satisfaction among female nurses pursuing a further degree in China. Int J Nurs Pract. 2022;28(5):e13014. doi: 10.1111/ijn.13014 34515390

[49] Goong H, Xu L, Li CY. Effects of work-family-school role conflicts and role-related social support on burnout in Registered Nurses: a structural equation modelling approach. J Adv Nurs. 2016;72(11):2762–2772. 27221584 10.1111/jan.13029

[50] Cortese CG, Colombo L, Ghislieri C. Determinants of nurses’ job satisfaction: the role of work-family conflict, job demand, emotional charge and social support. J Nurs Manag. 2010;18(1):35–43. doi: 10.1111/j.1365-2834.2009.01064.x 20465727

[51] Yang Z, Qi S, Zeng L, Han X, Pan Y. Work-Family Conflict and Primary and Secondary School Principals’ Work Engagement: A Moderated Mediation Model. Front Psychol. 2020;11:596385. doi: 10.3389/fpsyg.2020.596385 33584432 PMC7876373

[52] Liu J, Lambert EG, Kelley T, Zhang J, Jiang S. Exploring the Association Between Work-Family Conflict and Job Involvement. Int J Offender Ther Comp Criminol. 2020;64(8):791–817. doi: 10.1177/0306624X19896463 31874569

[53] Chen S, Wu H, Sun M, Wei Q, Zhang Q. Effects of shift work schedules, compensatory sleep, and work-family conflict on fatigue of shift-working nurses in Chinese intensive care units. Nurs Crit Care. 2023;28(6):948–956. doi: 10.1111/nicc.12909 37078518

[54] Simunić A, Gregov L. Conflict between work and family roles and satisfaction among nurses in different shift systems in Croatia: a questionnaire survey. Arh Hig Rada Toksikol. 2012;63(2):189–197. doi: 10.2478/10004-1254-63-2012-2159 22728801

